# Understanding diabetic cheiroarthropathy: a focus on clinical presentation

**DOI:** 10.1093/jscr/rjae123

**Published:** 2024-03-08

**Authors:** Elijah M Persad-Paisley, Chanbin Lee, Reena A Bhatt

**Affiliations:** Division of Plastic Surgery, Warren Alpert Medical School of Brown University, Providence, RI 02903, United States; Division of Plastic Surgery, Warren Alpert Medical School of Brown University, Providence, RI 02903, United States; Division of Plastic Surgery, Warren Alpert Medical School of Brown University, Providence, RI 02903, United States

**Keywords:** diabetic cheiroarthropathy, hyperglycemia, diabetic hand syndrome

## Abstract

Diabetic cheiroarthropathy (DCA) is a relatively uncommon and underdiagnosed complication of poorly controlled diabetes. It is caused by non-enzymatic glycation of collagen that ultimately leads to microvascular damage and polyarticular stiffness. If diagnosed early, optimal management of serum glucose levels may lessen joint stiffness and prevent microvascular and macrovascular complications associated with diabetes mellitus. We review the case of a 55-year-old male with type 2 diabetes mellitus who was diagnosed with DCA after complaints of chronic joint stiffness and immobility.

## Introduction

Diabetes mellitus is associated with musculoskeletal disorders of the hand, collectively referred to as diabetic hand syndrome. Diabetic cheiroarthropathy (DCA) is the condition in which there is: limited hand joint mobility, fixed flexion contracture at the metacarpophalangeal (MCP), proximal interphalangeal (PIP), and distal interphalangeal (DIP) joints; and palmar and dorsal surface sclerotic changes [1]. DCA occurs most frequently among patients with suboptimal glycemic control [1, 2]. We report a case of a DCA patient who presented to the plastic surgery hand clinic.

## Case report

A 55-year-old male with type 2 diabetes mellitus (T2DM) presented to the hand clinic in May 2023 for evaluation of a left-hand ulceration. He suffered from an occupational puncture injury. A superficial wound on the hand developed at the puncture site and did not appear to heal thereafter. His primary care physician recommended hand specialist follow-up.

**Figure 1:**
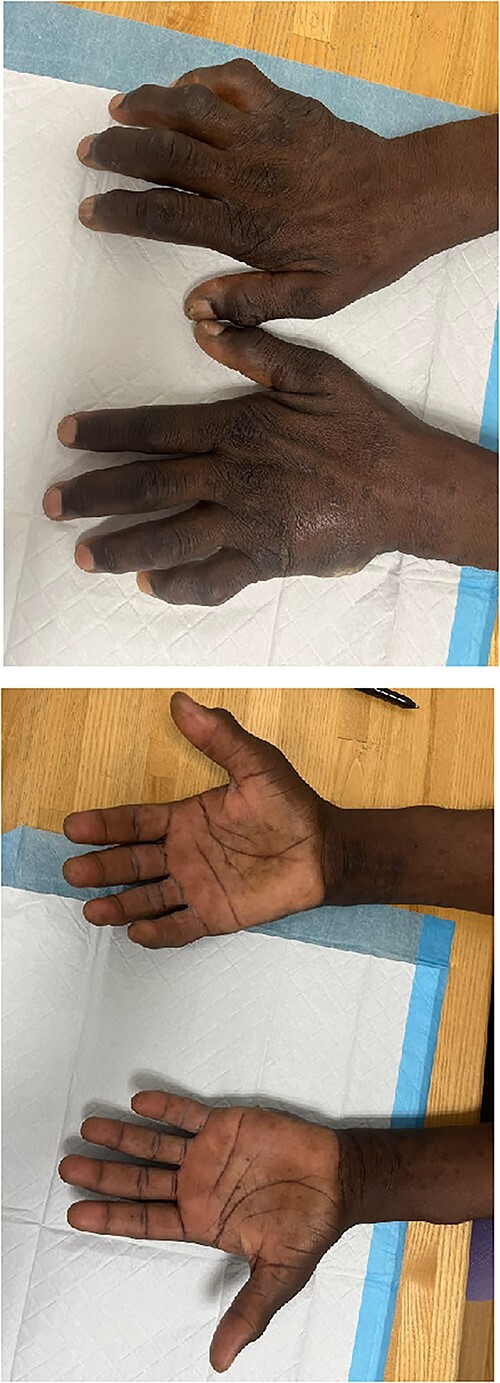
Images depicting patient hands after being instructed to lay flat on a level surface. Dorsal (A) and volar (B) aspects of the bilateral hands demonstrating marked joint enlargement and prominent flexion of bilateral MCP joints (right > left).

Upon evaluation in the hand clinic, the soft tissue of the dorsal hands appeared diffusely thickened bilaterally. Severe intrinsic muscle atrophy was noted. There were flexion contractures of the bilateral second and fifth interphalangeal (IP) joints. Decreased thumb opposition, IP joint extension, adduction and abduction of the first, second, fourth, and fifth digits were present bilaterally. Percussion at the bilateral cubital and carpal tunnels elicited paresthesias at the volar ulnar and median aspects of the hands, respectively. Application of pressure to the carpal tunnel between the hypothenar and thenar eminences with the hand in slight flexion produced pain and numbness bilaterally. The smallest distance at which two points of pressure could be detected was >10 mm over the median and ulnar aspects of the right hand, >10 mm on the ulnar aspect of the left hand, and equal to 6 mm over the median aspect of the left hand. Joint enlargement was present at the IP joints bilaterally. The hands were non-tender. An ulcer was present on the ulnar aspect of the left hand, lateral to the fifth metacarpal bone. Mild underlying induration was present without erythema, overlying eschar, or expressible drainage (Fig. 1A–B).

In April 2022, the patient was evaluated by his primary care physician for ongoing hand swelling, arthralgias, and a nonspecific rash. He received an extensive rheumatologic workup, which was unremarkable. There was a slightly elevated erythrocyte sedimentation rate (28 mm/hr [normal: 0–20 mm/hr]). Hemoglobin A1c (HbA1c) was 13.1% and the microalbumin-to-creatinine ratio was 589 mg/g (normal: 0–19 mg/g). X-ray imaging was unrevealing. He was prescribed metformin and neutral protamine Hagedorn (NPH) insulin.

In May 2023, follow-up magnetic resonance imaging (MRI) of the left hand demonstrated: erosions at all MCP joints, third and fifth PIP joints, first IP joint, and capitate bone; joint space loss of the fifth IP joint with mild surrounding soft tissue thickening; capsular edema of the third and fifth PIP joints; and enhancement of the tendons in the first extensor compartment. Based on the patient’s history, exam, imaging, and laboratory values in the setting of a chronic hyperglycemic state, a diagnosis of DCA and neuropathy was made. The risks of poorly controlled diabetes and subsequent therapeutic options were discussed with the patient, with glycemic control being of paramount importance. He was instructed to follow up with his endocrinologist and receive close monitoring of his serum glucose. As of November 2023, the patient’s HbA1c was 10.9%. He is scheduled to undergo an unspecified toe amputation.

## Discussion

DCA occurs in up to 40% of T2DM patients with poor glycemic regulation [3, 4]. While the risk of DCA occurrence has been shown to increase with higher HbA1c levels, the development of limited hand joint mobility has also been reported to occur early in the course of diabetic disease [5].

The development of DCA is multifactorial, and several mechanisms have been identified. Persistent hyperglycemia facilitates non-enzymatic glycation of type II collagen, enabling cross-linkage of collagen fibers, thereby rendering them resistant to mechanical and enzymatic degradation [6]. The proliferation of increasingly tensile collagen in periarticular tissue can therefore lead to polyarticular stiffness. In addition to collagenous biochemical changes, on-going microangiopathic transformation of the skin and subcutaneous vessels can lead to low-grade ischemia of the tissues. The chronic hypoxic conditions and subsequent tissue necrosis results in fibrosis, which results in a ‘waxy’ skin appearance.

### Clinical features and diagnosis

Early recognition of DCA is critical in preventing further musculoskeletal and vascular complications [7]. The diagnosis of DCA is clinical. Patients generally present with painless stiffness and contractures of the hand that impair grip strength and fine motor control. PIP and MCP joint contractures may be present on physical exam. Thickened, waxy skin (most pronounced on the dorsum of the fingers) is present in some cases. The DIP joints, wrist, and axial skeleton are less commonly affected [8].

Two simple bedside tests are commonly used in the diagnosis of DCA. The ‘prayer sign’ can be made by asking the patient to join the palmar faces of the hands and fingers while holding the wrists in slight dorsiflexion. The test is considered positive when the MCP, PIP, or both sets of joints do not make full contact at the palmar surface [9]. The ‘tabletop sign’ can be elicited and is considered positive if the palmar aspect of the hand is unable to lay flat on a level surface [10].

Imaging is not routinely used to diagnose DCA. However, ultrasonography can detect thickening of the flexor tendon sheets and MRI may reveal flexor digitorum tendon thickening and tendon sheath enhancement, as was the case with our patient [11, 12].

### Clinical management

DCA appears to be an irreversible condition with no clear consensus regarding treatment modalities. Optimal glycemic control is frequently cited as the mainstay treatment for DCA [1, 7, 13]. Improved glycemic control is thought to potentially alleviate limited joint mobility [13] with some authors even noting complete reversal of symptoms [14, 15]. However, these claims have been minimally substantiated. Physical therapy, anti-inflammatory medications, and corticosteroid injections have been proposed for symptomatic relief, though the benefits remain unclear.

## Conclusion

Patients presenting with joint stiffness and immobility should be evaluated for diabetes mellitus and DCA if rheumatologic work-up is unremarkable. The simple clinical diagnosis of DCA can prevent worsening of musculoskeletal diabetic complications.
